# Distinct roles of the RasGAP family proteins in *C. elegans* associative learning and memory

**DOI:** 10.1038/srep15084

**Published:** 2015-10-15

**Authors:** M. Dávid Gyurkó, Péter Csermely, Csaba Sőti, Attila Steták

**Affiliations:** 1 Semmelweis University, Department of Medical Chemistry, Budapest, Üllői út 26 1085, Hungary; 2 University of Basel, Transfaculty Research Platform Molecular and Cognitive Neurosciences, Birmannsgasse 8, 4055 Basel, Switzerland; 3 University of Basel, University Psychiatric Clinics, Wilhelm Klein-Strasse 27, 4055 Basel, Switzerland

## Abstract

The Ras GTPase activating proteins (RasGAPs) are regulators of the conserved Ras/MAPK pathway. Various roles of some of the RasGAPs in learning and memory have been reported in different model systems, yet, there is no comprehensive study to characterize all *gap* genes in any organism. Here, using reverse genetics and neurobehavioural tests, we studied the role of all known genes of the *rasgap* family in *C. elegans* in associative learning and memory. We demonstrated that their proteins are implicated in different parts of the learning and memory processes. We show that *gap-1* contribute redundantly with *gap-3* to the chemosensation of volatile compounds, *gap-1* plays a major role in associative learning, while *gap-2* and *gap-3* are predominantly required for short- and long-term associative memory. Our results also suggest that the *C. elegans* Ras orthologue *let-60* is involved in multiple processes during learning and memory. Thus, we show that the different classes of RasGAP proteins are all involved in cognitive function and their complex interplay ensures the proper formation and storage of novel information in *C. elegans*.

The Ras family of small GTPase proteins plays a central role in many biological processes including cell proliferation, differentiation, cell migration, as well as in synaptic transmission and memory formation^1^,^2^,^3^,^4^. The activity of Ras is regulated by its guanine nucleotide-binding state. The active GTP-Ras slowly becomes inactive due to its intrinsic GTPase activity that hydrolyzes GTP to GDP. In addition, the activity of Ras is also regulated by two groups of proteins. Ras guanine exchange factors (GEFs) promote the dissociation of GDP from Ras and help the exchange of GDP to GTP, thus RasGEFs increase Ras activity. Contrarily, Ras GTPase activating proteins (RasGAPs) are negative regulators by enhancing the intrinsic GTPase activity of Ras. In contrast to vertebrates, the *C. elegans* genome encodes only three RasGAPs (*gap-1*, *gap*-2 and *gap*-3) each orthologous to respective major vertebrate subgroups^5^. *gap-1*, the representative of *rasal* subfamily, was originally described as a negative regulator of the LET-60/Ras pathway during vulval development^5^,^6^. *gap-2*, the homolog of the *syngap* subfamily, was shown to suppress the larval lethality of the *let-23*/EGF receptor and *let-60* hypomorphic mutants^7^. Finally, *gap-3* corresponding to the vertebrate *rasa1* (also known as p120^RasGAP^), is a predominant negative regulator of *let-60* signaling during germ cell development^5^. Interestingly, deletion of any single *gap* gene has no visible phenotype in *C. elegans*. In addition to the conservation of *rasgap* genes in nematodes, *C. elegans* also exhibits different forms of associative learning, and short-term and long-term memory^8^,^9^,^10^. Thus, *C. elegans* represents an ideal system to understand the role and possible interplay of the different *rasgap* genes during learning and memory.

In the current study, we investigated the roles of th*e gap* gene family members in locomotion, olfaction, associative learning and memory formation. Our results indicate that *gap-1* contributes to olfaction and associative learning; *gap-2* is involved in short-term and long-term associative memory with a minor, redundant role in associative learning, while *gap-3* plays a major role in olfaction, short- and long-term memory, and act redundantly with *gap-2* during associative learning.

## Results

### *C. elegans* RasGAPs are dispensable for chemotaxis to olfactory cues

Aversive olfactory associative learning and memory rely on chemotaxis because environmental cues are acquired by olfaction. To test the role of the *gap* genes in these processes, we first analysed the chemotaxis towards three chemoattractants (diacetyl, benzaldehyde, and isoamyl-alcohol) of single *gap* mutant worms or of double mutant animals carrying mutations in *gap* genes in different combination. Chemotaxis assays were conducted with chemicals at two different concentrations in order to be able to identify even mild sensory deficits of the mutant strains. The volatile compounds used in the assay represent bacterial metabolic molecules that can be nutriments, therefore, induce attraction^11^.

**Figure 1:**
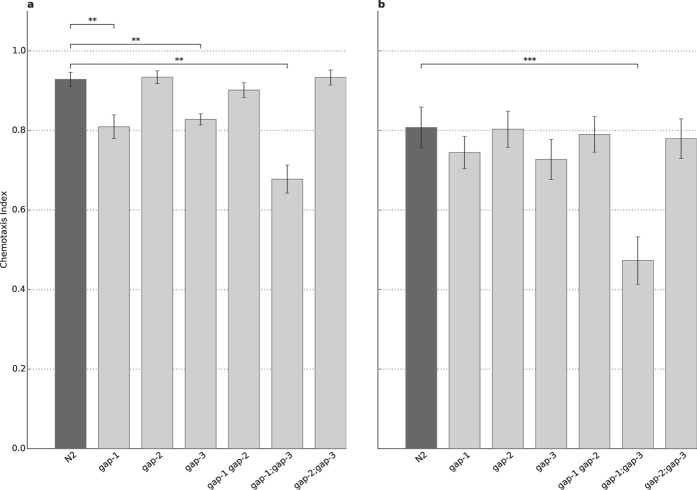
Involvement of various RasGAP isoforms in chemotaxis towards diacetyl. (**A**) Chemotaxis to 1:100 diluted diacetyl, attraction of N2 wild type (n = 33) and animals carrying the mutation(s) *gap-1(ga133)* (n = 9, p = 7.88 × 10^−3^), *gap-2(tm748)* (n = 18), *gap-3(ga139)* (n = 6, p = 2.45 × 10^−3^), *gap-1(ga133) gap-2(tm748)* (n = 9), *gap-1(ga133);gap-3(ga139)* (n = 7, p = 1,80 × 10^-3^) and *gap-2(tm748);gap-3(ga139)* (n = 15). (**B**) Chemotaxis to 1:1000 diluted diacetyl, attraction of N2 wild type (n = 31) and animals carrying the mutation(s) *gap-1(ga133)* (n = 31), *gap-2(tm748)* (n = 52), *gap-3(ga139)* (n = 31), *gap-1(ga133) gap-2(tm748)* (n = 24), *gap-1(ga133);gap-3(ga139)* (n = 29, p = 1.17 × 10^-14^) and *gap-2(tm748);gap-3(ga139)* (n = 36). Error bars indicate SD and asterisks indicate Bonferroni-corrected significant differences (**P < 0.01, ***P < 0.001).

We found that all the single *gap* mutants as well as most of the double mutant worms demonstrated an attraction to olfactory cues similar to the wild type across the conditions used. The *gap-2(tm748)*, *gap-3(ga139)*, *gap-1(ga133) gap-2(tm748)* and *gap-2(tm748);gap-3(ga139)* mutants showed normal chemotaxis towards diacetyl, benzaldehyde and isoamyl-alcohol at both high and low concentrations (Fig. 1 and Suppl. Fig. 1). Interestingly, *gap-1(ga133)* mutants demonstrated decreased attraction towards high concentration of diacetyl (p = 7.88 × 10^−3^) and both concentrations of isoamylalcohol (p = 2.14 × 10^−2^ for high and p = 1.57 × 10^−2^ for low concentration) while chemotaxis to benzaldehyde remained unaffected (Suppl. Fig. 1). The *gap-3(ga139)* mutant had a decreased attraction towards high concentration of diacetyl (p = 2.45 × 10^−3^), which was not observable in any other condition. The *gap-1(ga133);gap-3(ga139)* mutant had a strong chemosensory defect towards all compounds tested (Fig. 1 and Suppl. Fig. 1).

In summary, strains carrying *rasgap* mutations all respond to chemotactile stimuli. GAP-1 is involved in the chemosensation of isoamyl-alcohol and high concentration of diacetyl, while GAP-3 plays a role in the olfaction of high concentration of diacetyl only. For both single mutants we observed a statistically significant but only marginal decrease in the odor response. Futhermore, none of the mutations caused significant changes in chemosensation to low concentrations of diacetyl, and therefore these do not interfere with negative olfactory associative learning and memory tests. The only exception was the *gap-1(ga133);gap-3(ga139)* mutant, which had a chemosensory defect to both concentrations of all tested attractants.

### RasGAPs have no impact on locomotor behaviour and response to food

Similar to olfactory defects, impaired locomotion and lack of response to starvation could interfere with the assays addressing learning and memory functions. Therefore motility tests were conducted following three different feeding conditions to exclude locomotor or starvation response defects of the different *rasgap* single or double mutant strains.

**Figure 2:**
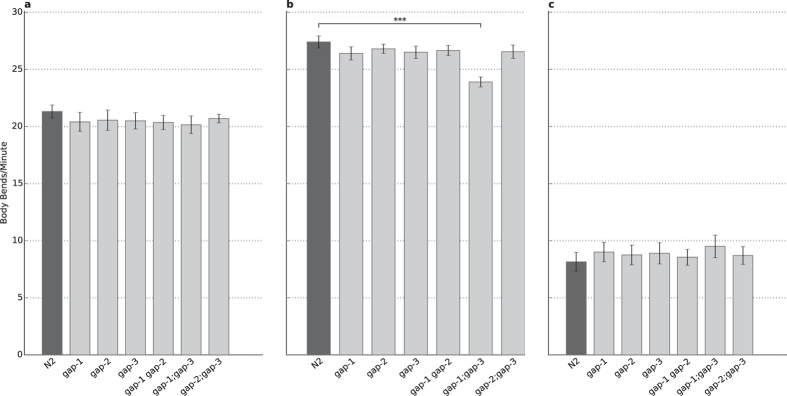
Locomotion is not affected by *gap* mutations. **(A)** Baseline feeding activity of *gap* mutant worms compared to the wild type N2. **(B)** Food searching activity of the N2 wild type and the *gap* mutants. The *gap-1;gap-3* double mutant shows decreased locomotion (p = 3.62 × 10^−13^). **(C)** *gap* mutations do not affect the feeding activity of the worms after one hour of starvation. n = 20 for each strain in each condition, error bars indicate SD and asterisks indicate Bonferroni-corrected significant differences (***P < 0.001).

We first tested the motility of well-fed worms on bacterial lawn to determine the baseline activity. Next we tested the food seeking behaviour by placing well-fed worms on empty plates. Finally, we investigated the starvation response of the different mutants by placing 1-hour-long starved worms on food source. No difference was observed in the mean body bends for fed (Fig. 2A) and starved (Fig. 2C) animals having *gap* mutations compared to the N2 reference strain. Food seeking animals showed similar behaviour except the *gap-1(ga133);gap-3(ga139)* mutant, which showed decreased motility (p = 2.54 × 10^−12^, Fig. 2B). A possible explanation of this difference is the deteriorated food searching motivation rooting in the chemosensory defect described above, because no locomotor defect was found in the ‘Fed’ and ‘Empty’ conditions.

From these experiments we concluded that locomotion is intact for the *gap-1(ga133)*, *gap-2(tm748)*, *gap-3(ga139)*, *gap-1(ga133) gap-2(tm748)*, *gap-2(tm748);gap-3(ga139)* strains in any experimental condition. The *gap-1(ga133);gap-3(ga139)* strain was excluded from further testing due to its chemosensory and possible locomotory defect.

### Complex regulation of learning and short-term associative memory by RasGAP proteins

In the previous experiments we tested all *gap* single and double mutants for defects that would interfere with negative olfactory associative learning. Since all *gap* single mutants and all *gap* double mutant worms (except the *gap-1;gap-3* mutants) showed no or minor defect in chemotaxis or motility, next the role of different RasGAPs during negative olfactory learning and short-term memory (STAM) was investigated^12^. Naïve worms are attracted to diacetyl, which can be turned into aversion during a 1-hour conditioning period that associates starvation with the previously attractive diacetyl. This association deteriorates over time, therefore the effectiveness of conditioning itself informs about learning, while the amount of deterioration characterizes the memory function.

**Figure 3:**
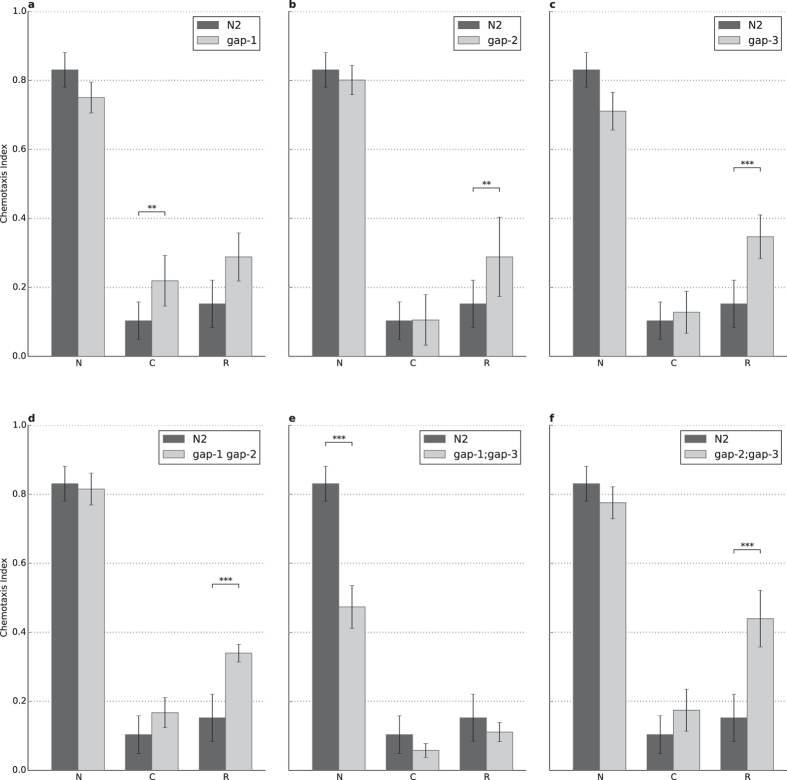
A complex interplay of RasGAPs is involved in associative learning and short-term memory. **(A)** *gap-1* mutation leads to a defect in learning (n = 23, p = 6.18 × 10^−3^) without short-term memory being affected. **(B**–**D,F)** Strong defect in short-term memory was observable for the (**B**) *gap-2* (n = 33, p = 1.34 × 10^−2^), (**C**) *gap-3* (n = 23, p = 2.75 × 10^−7^), (**D**) *gap-1 gap-2* (n = 15, p = 3.14 × 10^−12^) and (**F**) *gap-2;gap-3* (n = 21, p = 3.45 × 10^−7^) mutant animals without significant defect in learning. **(E)** The *gap-1;gap-3* double mutant could not be assessed due to its chemosensory defect (n = 24, p = 8.02 × 10^−13^). N: naïve, C: conditioned, R: recovered animals (see Materials and Methods for details). Error bars indicate SD and asterisks indicate significant differences (**P < 0.01, ***P < 0.001).

First, we tested all three single mutants and compared chemotaxis to diacetyl in naïve or conditioned worms. The combination of a 1-h starvation period in the presence of diacetyl (conditioning stimulus) dramatically reduced the attraction towards the chemoattractant in wild-type animals and in *gap-2* and *gap-3* single mutants to similar extent, but we found acquisition defects in the *gap-1(ga133)* (p = 0.00139) worms (Fig. 3 and Table 1). *gap-1* mutant worms also showed a significant memory defect when compared to wild types (p = 9.7 × 10^−6^), however, this may be due to the learning defect. To address that, we analyzed the rate of memory loss by comparing the difference between conditioned and 30 min delay attraction (also called recovery phase) to diacetyl in wild type and *gap-1* mutant worms and we found a statistically not significant difference between the genotypes. Thus, the memory difference was likely a consequence of the acquisition defect and this suggests that *gap-1* is playing a role in the learning process. In the case of the other two *gap* mutants, we observed a defect in both *gap-2* and *gap-3* only in memory (Fig. 3) and this suggests that *gap-2* and *gap-3* are both regulating memory consolidation.

Besides the single mutants we also tested the effect of simultaneous removal of multiple *rasgap*s. We found that the *gap-1(ga133) gap-2(tm748)* (p = 0.0016) and the *gap-2(tm748);gap-3(ga139)* double mutants (p = 1.5 × 10^−7^) displayed specific impairment of short-term memory (Fig. 3). Finally, the *gap-1(ga133);gap-3(ga139)* double mutant displayed a strong chemotaxis defect as described earlier and the strain could not be assessed further due to this sensory deficit (Fig. 3).

**Figure 4:**
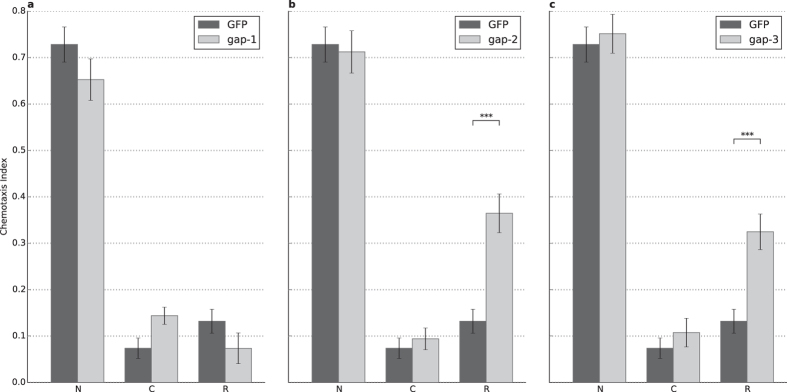
RNAi silencing of GAP-2 and GAP-3 phenocopy the mutant phenotypes. Negative conditioning assays were combined with RNA interference experiments using *eri-1(mg366);lin-15B(n744)* RNA sensitized worms, fed against (**A**) *gap-1 (n = 3)*, (**B**) *gap-2 (n = 13, p = 2.72 × 10*^*−7*^), and (C) *gap-3 (n = 9, p = 3.75 × 10*^*−5*^) dsRNA carrying bacteria. Dark grey represents the same strain fed with bacteria carrying an empty GFP marker dsRNA as reference. N: naïve, C: conditioned, R: recovered animals (see Materials and Methods for details). Error bars indicate SD and asterisks indicate significant differences (***P < 0.001).

**Figure 5:**
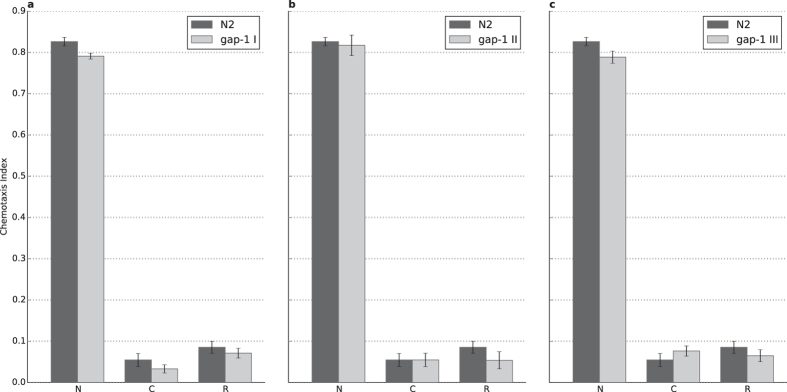
Re-introduction of *gap-1* gene rescues the loss-of-function *gap-1* phenotype. (**A**–**C**) Negative conditioning assays were performed with three independent *gap-1* rescue lines. Light grey bars represent the rescue lines and dark grey bars represent the reference N2 strain. n = 6 for all lines in each condition. N: naïve, C: conditioned, R: recovered animals (see Materials and Methods for details). Error bars indicate SD.

In order to exclude possible background mutations that could contribute to the observed phenotypes, we performed RNAi silencing of the different *rasgap*s in RNAi hypersensitive *eri-1(mg366);lin-15B(n744)* strain. As control, *eri-1(mg366);lin-15B(n744)* was fed with bacteria expressing GFP dsRNA (Fig. 4). Double stranded RNA targeting *gap-2* and *gap-3* mRNA caused a phenotype matching the *gap-2* and *gap-3* loss-of-function mutants, respectively (Fig. 4). In case of *gap-1* RNAi we did not observe this effect, probably due to insufficient RNAi silencing. Thus, we performed a rescue experiment by reintroducing the *gap-1* genomic fragment in *gap-1(ga133)* mutant worms and negative STAM was assessed in three parallel lines (see Materials and Methods for details). Genomic fragment encompassing the *gap-1* locus restored learning and memory to levels comparable to wild type in all three lines (Fig. 5). Thus, the observed phenotypes are attributable to the loss-of-function of various *rasgap* genes.

### Interplay of RasGAP proteins during long-term associative memory (LTAM)

Beside the role of RasGAPs in short-term memory, we also tested the impact of the different *C. elegans rasgaps* in long-term associative memory. LTAM was assessed by testing worms 16 and 24 hours after negative olfactory conditioning, as described previously^10^. In this experimental setup we used a reinforcement training consisting of three rounds of conditioning cycles. Briefly, starvation and diacetyl were associated for 1 hour, which turned the attraction towards diacetyl into aversion. Each conditioning step was followed by a feeding phase without diacetyl for 30 minutes to let the worms regenerate. Such rounds of conditioning and feeding were repeated 3 times. Using this training we could not detect a significant difference in acquisition in most *gap* mutant genotypes. A possible explanation of this phenomenon is that the naïve animals were conditioned 3 times in these assays compared to the single conditioning in assays testing STAM. Interestingly, the learning defect was still present in the *gap-2(tm748);gap-3(ga139)* double mutant strain (p = 1.24 × 10^−7^).

**Figure 6:**
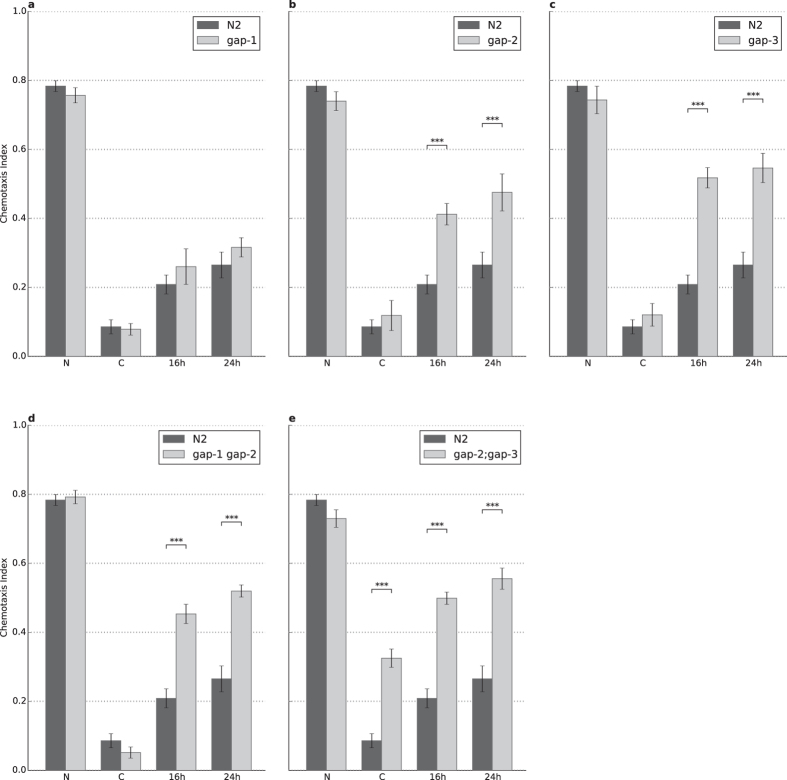
RasGAPs are involved in long-term associative memory. (**A**) gap-1(ga133) mutants show no significant defect in learning or in long-term associative memory (n = 5). (**B**) *gap-2(tm748)* (n = 12, p_16h_ = 5.61* × 10*^*−7*^, p_24h_ = 6.89* × 10*^*−5*^), (**C**) *gap-3(ga139)* (n = 15, p_16h_ = 9.33* × 10*^*−15*^, p_24h_ = 1.67* × 10*^*−10*^), (**D**) *gap-1(ga133) gap-2(tm748)* (n = 6, p_16h_ = 2.53* × 10*^*−4*^, p_24h_ = 5.18* × 10*^*−8*^) mutants all have long-term associative memory defect together with (**E**) *gap-2(tm748);gap-3(ga139)* (n = 9, p_16h_ = 3.57* × 10*^*−13*^, p_24h_ = 7.93* × 10*^*−7*^) mutants, which also display learning defect (p = 8.70* × 10*^*−7*^). The N2 wild type represents the reference on all charts. Naïve (N) animals were conditioned (C), then tested after 0.5 hour recovery (R), and 16 hours (16h) and 24 hours (24h) after conditioning (see Materials and Methods for details). Error bars indicate SD and asterisks indicate significant differences (***P < 0.001).

Strikingly, *gap-1(ga133)* did not show any impairment in learning or in long-term memory while all other two single mutants had a specific memory defect similar to their short-term associative memory phenotype (Fig. 6, Table 1). As shown in Fig. 6, remaining two double mutants*, gap-1(ga133) gap-2(tm748)* (p = 6.75 × 10^−7^) and *gap-2(tm748);gap-3(ga139)* (p = 2.00 × 10^−6^) had similarly strong long-term associative memory defects. In the *gap-1(ga133);gap-3(ga139)* double mutant strain, the chemotaxis defect prevented any assessment of learning and memory formation. Altogether, the *rasgap* genes are redundantly regulating long-term memory in nematodes.

### *let-60* is involved in the *gap*–related learning and memory phenotypes

**Figure 7:**
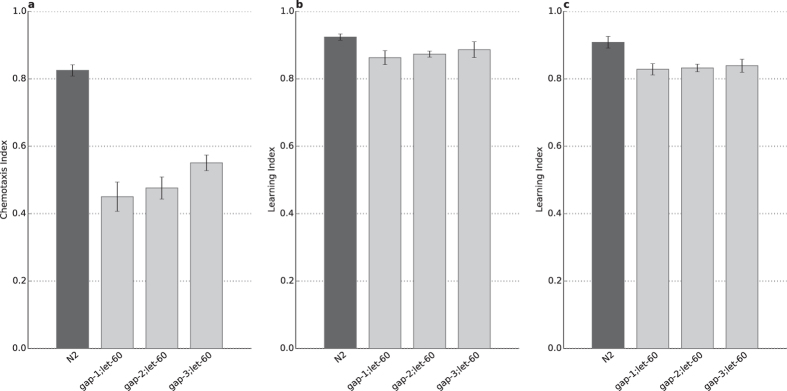
*let-60* is required for the *gap(lf)* learning and memory phenotypes . Naïve *gap(lf);let-60(n2021hf)* double mutants (**A**) were conditioned to assess learning (**B**) and short term associative memory (**C**). Naïve mutants are characterized by lowered chemotaxis index, e.g. chemosensory defect due to the *let-60(n2021hf)* mutation. Conditioned worms have no significant learning defect (**B**) and recovery phase has not revealed significant memory defect either (**C**). Both conditioned and recovery phases were assessed by calculating learning indices (LI = [CI_conditioned_ – CI_naïve_]/CI_naïve_) to ensure comparability with the N2 wild type.

RasGAPs are known to increase the intrinsic GTPase activity of LET-60 and by that they regulate different biological processes in *C. elegans*^13^. Based on these earlier observations we hypothesized that *gap* mutations combined with a reduction of function *let-60* mutation would result in a learning and memory phenotype similar to the wild type. The *let-60* mutation itself causes a strong chemosensory defect^14^, which was in part restored in all *gap;let-60* double mutant. Furthermore, in agreement with our assumptions, we found that the *let-60(n2021)* hypomorph mutation restored the learning and memory defects observed in the *rasgap* mutants (Fig. 7). This suggests that all three *rasgap*s act at least in part through *let-60* to regulate learning and memory in *C. elegans*.

## Discussion

Here, we investigated the role of each member of the RasGAP family in the learning and memory process in *C. elegans*. We found that all RasGAP forms are regulating cognitive functions, and strikingly, they exhibit specific roles during learning and memory. While the exact molecular machinery connecting RasGAPs to learning and memory formation in *C. elegans* is unclear yet, our results show that RasGAPs are likely involved in multiple distinct processes during acquisition and storage of new informations.

In *C. elegans*, synaptic cytoskeletal reorganization is widely accepted as a form of synaptic plasticity, and was suggested recently as a molecular process of forgetting^15^. Fast activation and inactivation of Ras is important for the olfactory behaviour^16^, although the negative feedback loop for inactivation has not been elucidated yet.

In humans, analysis of molecular signaling networks revealed potential cross-talks between the Ras/MAPK pathway and the possible cytoskeletal rearrangments^17^, which is further supported by the earlier finding that RasGAPs control Rho-mediated cytoskeletal reorganization^18^. In rat hippocampal neurons, synaptic Ras GTPase activating protein (SynGAP), an orthologue of *C. elegans* GAP-2, can be phosphorylated by the calcium/calmodulin-dependent protein kinase II (CaMKII) upon long-term potentiation induction. This leads to the dispersion of SynGAP from the dendritic spines^19^. Ras family proteins, the major effectors downstream of GAPs, have also been associated with Ca^2 + ^-dependent synaptic crosstalk after NMDA receptor activation in rat hippocampal slices^20^. Neurofibromin 1 (NF1), a GAP orthologue not found in *C. elegans* yet, can also inactivate Ras in rat hippocampal dendritic spines^21^. In mouse model systems, homozygous knock-out of *syngap* leads to postnatal lethality, while heterozygous mice exhibit specific defects in hippocampal long-term potentiation and glutamate receptor trafficking, although the molecular relation between these two processes is difficult to assess at phenotypic level^22^. In a mouse schizophrenia model, reduced expression of SynGAP leads to non-habituating mice showing persistent hyperactivity, lack of social memory, impaired fear conditioning and working memory, probably due to defected interaction between SynGAP and NMDA receptor^23^. Neurofibromin 1 also regulates GABA release, long-term potentiation and learning in mice^24^. Furthermore, in zebrafish, loss of Neurofibromin 1 results in learning and memory defects^25^. Altogether, these findings suggest a) a conserved role for different RasGAPs in learning and memory, b) a significant role for SynGAP in signal transmission during learning and memory formation. In addition to the NF1 and SynGAPs we show here that RASAL and p120^RasGAP^ subfamily members are also regulating learning and memory and that different RasGAP subclasses are redundantly modulating different parts of the acquisition and storage processes.

Furthermore, our results are in good agreement with clinical findings. Rasopathies are human pathological conditions associated with germline mutations of the Ras/MAPK pathway^26^,^27^. The overlapping phenotypic features are developmental and cutaneous abnormalities, predisposition to malignancies and varying degree of neurocognitive impairment including learning disability. Rasopathies involving RasGAPs are neurofibromatosis type 1 (NF1, also known as von Recklinghausen disease)^28^,^29^ and capillary malformation – arteriovenous malformation syndrome (CM–AVM)^30^, caused by germline mutations in the *nf1* and *rasa1* genes, respectively. The exact molecular mechanisms underneath the symptoms are poorly understood yet.

In conclusion, we characterized all three known genes of the *gap* gene family in *C. elegans* to show that *gap* genes are involved in olfaction, associative learning, short- and long-term memory formation. Our results are in good agreement with the characteristics known partially from other model systems, and are in accordance with the clinical features of Rasopathies, further supporting that the molecular mechanisms of learning, memory and the role of the Ras/MAPK pathway in these are well conserved. This work also opens perspectives for neurocognitive and neurobehavioural studies of RasGAPs in *C. elegans*.

## Materials and Methods

The reagents were obtained from Sigma (Sigma-Aldrich, St Louis, MO) unless otherwise indicated. Standard methods were used for maintaining and manipulating *C. elegans* strains^31^. Worms were grown at 20 °C on *E. coli* OP50 bacteria. Behavioural tests were performed with well fed, 1-day-old adult worms. The *C. elegans* Bristol strain, variety N2 was used as wild type reference otherwise indicated. *C. elegans* strains used were: *gap-1(ga133)*, *gap-2(tm748)*, *gap-3(ga139)*, *gap-1(ga133) gap-2(tm748)*, *gap-1(ga133);gap-3(ga139)*, *gap-2(tm748);gap-3(ga139)*, *let-60(n2021)*, and *eri-1(mg366);lin-15B(n744).*

For the *gap-1* rescue, the fosmid clone WRM0629aG09 was digested with AvrII/SbfI restriction endonucleases and the 9.5 kb fragment encompassing the *gap-1* gene was microinjected at a concentration of 100 ng/μl into both arms of the syncytial gonads of *gap-1(ga133)* worms as described earlier^32^. Sur-5::dsRed at 10 ng/μl concentration was coinjected as transformation marker.

### RNA interference (RNAi)

RNAi was performed with *eri-1(mg366);lin-15B(n744)* worms as previously described^33^. Animals in L3 stage were placed for 1 day on NGM plates containing 5 mM Isopropyl-D-thiogalactopyranoside (IPTG) and HT115(DE3) *E. coli* strain producing dsRNA against *gap-1*, *gap-2* and *gap-3,* respectively, as described^34^,^35^. Bacteria containing vector with GFP were used as control in all RNA interference experiments.

### Motility assay

Motility was characterized by the number of body bends per minute as described earlier^36^,^37^. Briefly, well fed single young adult worms were transferred onto seeded NGM plates (baseline activity), onto empty plates (food searching activity) or animals were starved for 1 hour and transferred to seeded NGM plates (feeding activity). Body bends were counted for 1 minute after a 2-minute resting phase. At least 20 animals were recorded per condition and per genotype.

### C. elegans behavior assays

Chemotaxis to different compounds was assessed as described previously^38^. Briefly, worms were washed three times in CTX buffer (5 mM KH_2_PO_4_/K_2_HPO_4_ pH 6.0, 1 mM CaCl_2_, 1 mM MgSO_4_) and were allowed to settle down by gravity. 50 to 200 worms were placed to the middle of 10 cm CTX test plates (5 mM KH_2_PO_4_/K_2_HPO_4_ pH 6.0, 1 mM CaCl_2_, 1 mM MgSO_4_, 2% agar). Worms were given a choice between a spot of attractant (diacetyl, benzaldehyde and isoamylalcohol) in ethanol at the indicated dilutions with 20 mM sodium-azide and a counter spot with ethanol and sodium-azide. The distribution of the worms over the plate was determined after 1 hour and the chemotaxis index was calculated as described earlier^38^.

Negative olfactory associative conditioning was performed with modifications of the original protocol^12^. For the conditioning, 1 hour long starvation was coupled with 2 μl of undiluted diacetyl dropped on the lid of 10 cm CTX plates. A subpopulation of worms was washed for half an hour in CTX buffer after conditioning to allow recovery. Chemotaxis of naïve, conditioned and recovery worms were tested with diluted diacetyl (1:1000) as described above.

Long-term associative memory assays were performed as described earlier^10^. Briefly, worms were conditioned three times by repeating the cycles of conditioning described above and by allowing the worms to regenerate for 30 mins in presence of food after each conditioning. Memory function was assessed after 16 and 24 hours as described for the negative olfactory associative conditioning.

Learning index was calculated as the difference of chemotaxis indices of conditioned and naïve worms normalized by the chemotaxis index of naïve animals:


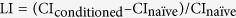


### Computational tools and statistics

The scripting language Python 3.4, the numerical library numpy 1.8 and the plotting library matplotlib 1.4.1 were used to analyze and visualize the experimental results. Welch’s test^39^, as implemented in the statistical module of SciPy 0.13.3, was used to calculate statistical significances. P-values always refer to results of two-tailed tests, in multiple comparisons p-values are always Bonferroni corrected; *p ≤ 0.05, **p < 0.01, ***p < 0.001. Two-way ANOVA with Bonferroni corrected post-hoc t-test was used for interaction analysis of learning and memory assays, these tests were performed with the R project (http://www.r-project.org/). Error bars represent standard deviation. All computational tools are open source and were designed and implemented by experts to follow the best practices and to ensure scientific reproducibility.

## Additional Information

**How to cite this article**: Gyurkó, M. D. *et al.* Distinct roles of the RasGAP family proteins in *C. elegans* associative learning and memory. *Sci. Rep.* **5**, 15084; doi: 10.1038/srep15084 (2015).

## Supplementary Material

Supplementary Information

## Figures and Tables

**Table 1:** Phenotypic features of *gap-1(ga133)*, *gap-2(tm748)*, *gap-3(ga139)*, *gap-1(ga133) gap-2(tm748)*, *gap-1(ga133);gap-3(ga139)*, *gap-2(tm748);gap-3(ga139)* strains, respectively, regarding chemosensation of diacetyl, motility, learning, short-term associative memory (STAM) and long-term associative memory (LTAM).

	Chemosensation	Motility	Learning	STAM	LTAM
*gap-1(ga133)*	Intact	Intact	Defect	Intact	Intact
*gap-2(tm748)*	Intact	Intact	Intact	Defect	Defect
*gap-3(ga139)*	Intact	Intact	Intact	Defect	Defect
*gap-1(ga133) gap-2(tm748)*	Intact	Intact	Intact	Defect	Defect
*gap-1(ga133);gap-3(ga139)*	Defect	Intact*	N/A	N/A	N/A
*gap-2(tm748);gap-3(ga-139)*	Intact	Intact	Defect	Defect	Defect

Defect = the strain behaves significantly different compared to the N2 wild-type reference. Intact = no significant difference. N/A: not assessed.

^*^The baseline fed and feeding locomotory rate of the *gap-1(ga133); gap-3(ga139)* line are intact. The difference of the food searching activity is probably due to a chemosensory defect.
