# Supplementary material for: Distinct roles of the RasGAP family proteins in C. elegans associative learning and memory
Source: Sci Rep. 2015 Oct 15;5:15084. doi: 10.1038/srep15084 (PMC4606830; doi:10.1038/srep15084)
Supplement: Supplementary Information [file srep15084-s1.pdf]

Distinct roles of the RasGAP family proteins in *C. elegans* associative learning and memory

Gyurkó, M. Dávid[1A]; Csermely, Péter[1B]; Sőti, Csaba[1C*]; Steták, Attila[2, 3 *]

[1] Semmelweis University, Department of Medical Chemistry

[2] University of Basel, Transfaculty Research Platform Molecular and Cognitive Neurosciences, Birmannsgasse 8, 4055 Basel, Switzerland

[3] University of Basel, University Psychiatric Clinics, Wilhelm Klein-Strasse 27, 4055 Basel, Switzerland

[*] Corresponding authors, e-mail: a.stetak@unibas.ch and soti.csaba@med.semmelweis-univ.hu

[A] ORCID: http://orcid.org/0000-0002-2997-9799

[B] ORCID: http://orcid.org/0000-0001-9234-0659

[C] ORCID: http://orcid.org/ 0000-0002-4057-7678

Supplementary Material

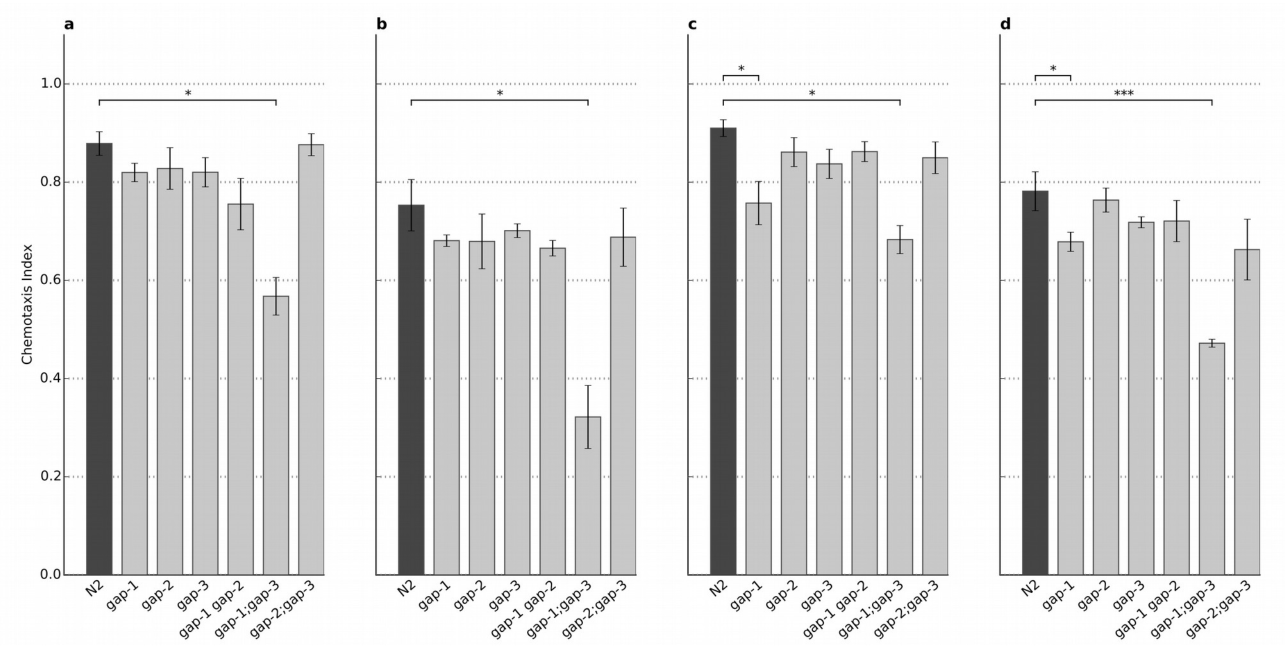

Supplementary Figure S1. GAP1 and GAP3 play redundant roles in the chemosensation of benzaldehyde, and individual roles in the chemosensation of isoamylalcohol.

(**a**, **b**) Chemotaxis of the *gap* mutants to high and low concentrations of benzaldehyde. *gap-1(ga133);gap-3(ga139)* mutants show significant chemotaxis defect both in case of (**a**) 1:100 diluted benzaldehyde (n=6, p=1.74×10^{-2}) and (**b**) 1:1000 diluted benzaldehyde (n=6, p=2.35×10^{-2}). (**c**, **d**) Chemotaxis to high and low concentrations of isoamylalcohol. (**c**) In case of 1:100 diluted isoamylalcohol, both gap-1(ga133) (n=9, p=2.14×10^{-2}) and *gap-1(ga133);gap-3(ga139)* (n=6, p=1.83×10^{-2}) has a significant chemotaxis defect. (**d**) Impaired chemosensorium was observed for 1:1000 diluted isoamylalcohol for both *gap-1(ga133)* (n=6, p=1.57×10^{-2}) and *gap-1(ga133);gap-3(ga139)* (n=6, p=6.46×10^{-13}).
The N2 wild type served as reference for all chemotaxis tests. Error bars indicate STD and asterisks indicate Bonferroni-corrected significant differences (* < 0.05, *** < 0.001).

Compared Conditions	Stat. Type	Df	Stat. Val.	Pbonf
WT_N vs gap-1_N	t	25.98704618	-4.261100464	0.000944744
WT_C vs gap-1_C	t	27.7270287	3.511601173	0.00617734
WT_R vs gap-1_R	t	32.11672727	4.156266923	0.000896519
(R-C) WT vs gap-1	F	-1,242	0.231274252	1
WT_N vs gap-2_N	t	44.89971023	-2.261224639	0.114552681
WT_C vs gap-2_C	t	40.5782204	0.085006932	1
WT_R vs gap-2_R	t	38.12634462	3.12885873	0.013429645
(R-C) WT vs gap-2	F	-1,259	10.21201408	0.006273259
WT_N vs gap-3_N	t	28.33330681	-5.475656315	2.92E-05
WT_C vs gap-3_C	t	34.0570314	0.90922098	1
WT_R vs gap-3_R	t	38.73600972	6.64217682	2.75E-07
(R-C) WT vs gap-3	F	-1,246	18.53696054	9.63E-05
WT_N vs gap-1;gap-2_N	t	16.06182133	-0.909958445	1
WT_C vs gap-1;gap-2_C	t	18.76862878	2.38818578	0.110441501
WT_R vs gap-1;gap-2_R	t	48.74725126	9.608492122	3.14E-12
(R-C) WT vs gap-1;gap-2	F	-1,225	6.779563311	0.039337786
WT_N vs gap-1;gap-3_N	t	26.08150575	-13.69916343	8.02E-13
WT_C vs gap-1;gap-3_C	t	101.5292101	-3.305559728	0.005243203
WT_R vs gap-1;gap-3_R	t	91.91593392	-2.296389462	0.095703813
(R-C) WT vs gap-1;gap-3	F	-1,244	0.013795593	1
WT_N vs gap-2;gap-3_N	t	24.1555002	-2.864899166	0.034008531
WT_C vs gap-2;gap-3_C	t	26.91506343	2.413441326	0.091533043
WT_R vs gap-2;gap-3_R	t	25.93676897	7.341879096	3.45E-07
(R-C) WT vs gap-2;gap-3	F	-1,238	24.56346286	5.46E-06

Supplementary Table 1. The statistical results of the post-hoc tests for short term associative memory assays.

Post-hoc t-tests were performed after the two-way analysis of variance to identify any interaction patterns caused by the *gap* mutations in learning and short term associative memory. F-tests were performed to compare the statistical distribution of the

measurements.

WT: wild-type

N: naïve, C: conditioned, R: recovery

Stat. Type: The type of the calculated statistics, t = t-test, F: F-test

Df: Degree of Freedom

Stat. Val: The result of the statistical calculation

Pbonf: Bonferroni-corrected P value.
